# “Biophilic Cities”: Quantifying the Impact of Google Street View-Derived Greenspace Exposures on Socioeconomic Factors and Self-Reported Health

**DOI:** 10.1021/acs.est.1c01326

**Published:** 2021-06-23

**Authors:** Anna C. O’Regan, Ruth F. Hunter, Marguerite M. Nyhan

**Affiliations:** † Discipline of Civil, Structural & Environmental Engineering, School of Engineering & Architecture, University College Cork, Cork, Ireland; ‡ MaREI Centre for Energy, Climate & Marine and Environmental Research Institute, University College Cork, Cork, Ireland; § Centre for Public Health, Queen’s University Belfast, Belfast BT12 6BA, Northern Ireland, United Kingdom; ∥ Harvard T.H. Chan School of Public Health, Harvard University, Boston, Massachusetts 02215, United States

**Keywords:** urban greenspace, urban analytics, Google Street View, normalized difference vegetation index, population-weighted exposure, public health

## Abstract

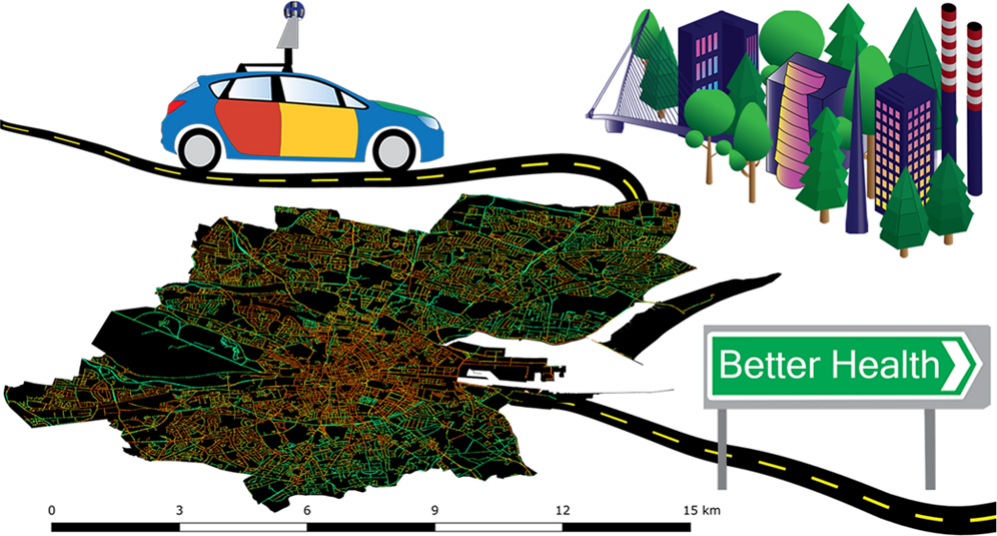

According to the biophilia hypothesis, humans have evolved to prefer natural environments that are essential to their thriving. With urbanization occurring at an unprecedented rate globally, urban greenspace has gained increased attention due to its environmental, health, and socioeconomic benefits. To unlock its full potential, an increased understanding of greenspace metrics is urgently required. In this first-of-a-kind study, we quantified street-level greenspace using 751 644 Google Street View images and computer vision methods for 125 274 locations in Ireland’s major cities. We quantified population-weighted exposure to greenspace and investigated the impact of greenspace on health and socioeconomic determinants. To investigate the association between greenspace and self-reported health, a negative binomial regression analysis was applied. While controlling for other factors, an interquartile range increase in street-level greenspace was associated with a 2.78% increase in self-reported “good or very good” health [95% confidence interval: 2.25–3.31]. Additionally, we observed that populations in upper quartiles of greenspace exposure had higher levels of income and education than those in lower quartiles. This study provides groundbreaking insights into how urban greenspace can be quantified in unprecedented resolution, accuracy, and scale while also having important implications for urban planning and environmental health research and policy.

## Abstract 2

Urban greenspace is quantified in unprecedentedly high spatial resolution and scale using Google Street View imagery and computer vision methods. Exposure to urban greenspace is associated with improved health in urban residents.

## 1 Introduction

Urbanization is occurring at an unprecedented rate worldwide, a trend that poses immense challenges to urban sustainability, livability, and public health. Currently, 55% of the world’s population live in urban areas, which is set to increase to 70% by 2050.^1^ There is tremendous opportunity to counteract some of the negative impacts of urbanization on human health and well-being,^2^ with positive environmental exposures such as urban greenspace.^3−7^ Urban greenspace refers to natural environments, parks, and recreational spaces; green infrastructure including walking and cycling lanes; and natural vegetation including tree-lined streets, shrubs, gardens, lawns, green walls, and green roofs.

In environmental epidemiological literature, increased exposure to greenspace has been associated with lower mortality rates,^3,4,7^ greater life satisfaction,^8^ and improved mental health and well-being.^9,10^ Additionally, urban residents are more likely to engage in physical activity, a key factor for noncommunicable disease prevention, in greenspace environments.^11,12^ In the current technological era, promoting outdoor activity among younger generations through enhanced greenspace provision is extremely important. Dadvand et al.^13^ found that children living in close proximity to greenspace had reduced screen time and lower obesity rates. Urban greenspace alleviates adverse environmental health impacts related to pollution and climate change^14−16^ as it mitigates air pollution, reduces noise pollution, and enhances the thermal environment.^17−20^

Urban greenspace has numerous social benefits including reduction in crime rates, increased perception of safety,^16^ and encouraging interaction between residents, leading to social cohesion.^2^ Greenspace enhances the esthetics of urban areas. It attracts people, investment, and development and is associated with higher property values.^21^ Such green gentrification can exclude socially and economically vulnerable residents due to social isolation or forcing them to relocate.^22^ Research has shown that greenspace provision is not spatially or socially equitably distributed.^23,24^ Therefore, reducing disparities in greenspace availability is important to prevent widening of health, environmental, and social inequalities.

Due to the Covid-19 pandemic, governments have implemented public health guidance including restricting travel and indoor gatherings.^25^ This further highlights the need for “safe, inclusive and accessible green and public spaces”.^26^ However, greenspace development is hindered by increased demand for residential, transport, commercial, and infrastructure developments.^27^ The successful management of fast-paced urbanization and urban greenspace is critical to ensure healthy and livable cities.

A major barrier to successful urban greenspace management is the lack of details in greenspace exposure maps including those used to examine socioeconomic and human health benefits of urban greenspace. Established methods for benchmarking greenspace, including questionnaires, field audits, and geographic information systems (GIS), have several limitations.^28,29^ Questionnaires and field audits are labor-intensive, time consuming, and limited to small study domains.^30^ GIS and satellite imagery offers objective and efficient aerial assessment of greenspace over large spatial scales. Established aerial greenspace metrics include the normalized difference vegetation index (NDVI), % greenspace, % street tree buffering, and distance to parks.^31^ These aerial views fail to capture street-level greenspace as experienced by humans on the ground. However, high-resolution street-level greenspace computed over large spatial scales is not routinely available.

Recent advances in street-level imagery availability, data processing, and computer vision methods have great potential for improving urban street-level greenspace assessment.^32−36^ By obtaining publicly available high-resolution Google Street View (GSV) images and applying computer vision methods, an accurate measure of visible greenspace can be achieved. Over 10 million miles of GSV images have been captured globally.^37,38^ Although studies have shown significant potential to advance greenspace exposure assessment, they have not quantified and compared urban street-level greenspace in extremely high spatial resolution for all major cities in an entire country. Furthermore, previous research has neither examined population-weighted exposure to greenspace nor examined how health and socioeconomic variables vary with these greenspace metrics within and among cities on a national scale.

To advance our understanding of urban greenspace and unlock its full socioeconomic and health benefit potential, we quantified urban street-level greenspace for three major Irish cities in unprecedented accuracy, spatial resolution, and scale using 751 644 GSV images and computer vision methods. Street-level greenspace was compared to NDVI. Following this, population-weighted exposures to these greenspace metrics were quantified. Finally, all greenspace metrics and population-weighted exposures were investigated in relation to socioeconomic and health variables.

## 2 Methods

### 2.1 Protocol and Study Domain

Three major Irish cities were included in our study, namely, Dublin city, Cork city, and Galway city. Located in the east, Dublin is the capital of Ireland with a population of 550 000.^39^ Cork city, located in the south of Ireland, has a population of 210 000 and an area of 187 km^2^.^39,40^ Galway city, located in the west, has a population of 80 000 and covers an area of 54 km^2^.^39,41^ Urban greenspace was quantified at street-level and overhead using GSV and satellite imagery, respectively, throughout our study domain. These greenspace metrics were examined with respect to socioeconomic and health measures, and associations between all greenspace metrics and self-reported “good and very good” health were then determined.

### 2.2 Quantifying Urban Greenspace Using GSV Imagery and Computer Vision Methods

**Figure 1:**
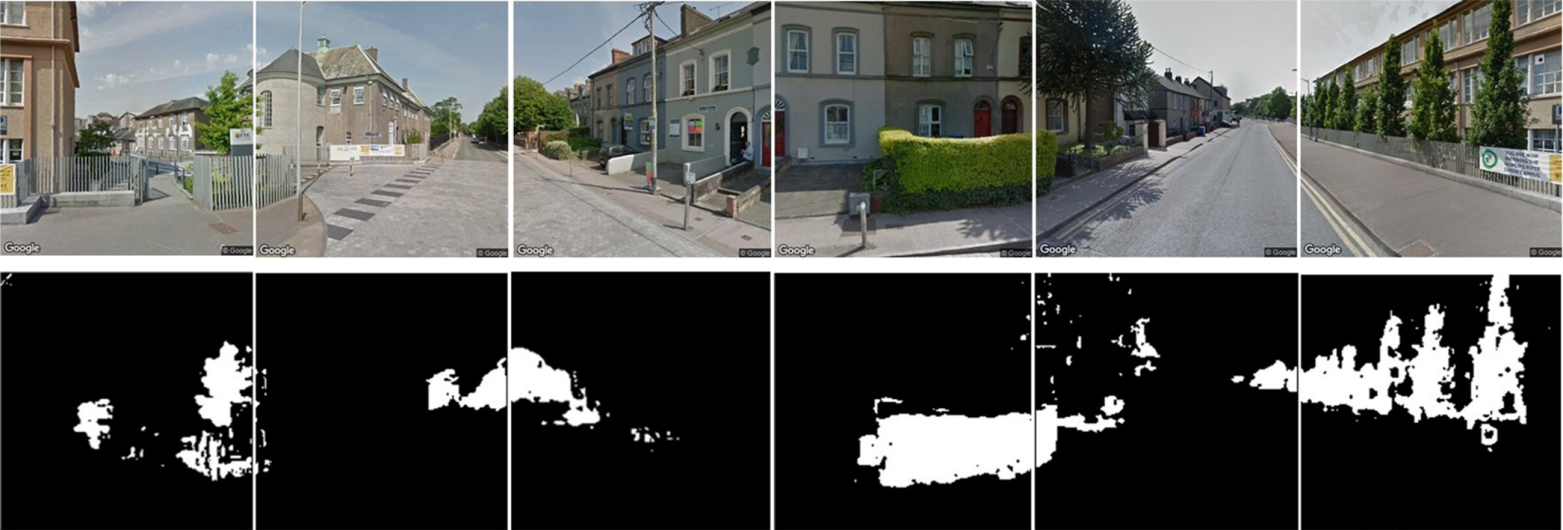
Street-level urban greenspace was quantified using 751 644 GSV imageries and computer vision methods in high spatial resolution (125 274 locations) on the entire road networks of three major cities in Ireland. Here, a six-image Google Street View panorama generated for a single point location in Cork city (top images) and its corresponding image segmentation and, thus, classification of green vegetation as white in each image (bottom images) are shown.

Street-level urban greenspace was quantified using 751 644 GSV images and computer vision methods in high spatial resolution (125 274 point locations) for Dublin, Cork, and Galway cities. For each city, global positioning system (GPS) points were generated every 50 m on a road network shapefile. Using a Google Application Programming Interface (API) key, metadata of the GSV panoramas, including an ID, date, latitude, and longitude, were collected for each point. For each point location, six GSV images were downloaded capturing different horizontal viewing angles (every 60°) (see Figure 1). Across the three cities, GSV images were captured from 2009 to 2019, with 65% of the images taken between 2017 and 2019. Based on the seasonal visual appearance of greenery in Ireland, only GSV images captured in March to October inclusive (“green” months) were used to determine greenspace; 125 274 locations met this criterion, resulting in 751 644 GSV images. Existing Python scripts were adopted and modified to generate point locations and to process the GSV images.^42^

To classify greenspace in each image, we adopted an object-based image analysis technique. Images were segmented into homogeneous polygons, which are physically meaningful, minimizing misclassification of green objects as greenspace.^43^ The Python module *pymeanshift* was used to perform image segmentation. The contrast between greenspace pixels and nongreenspace pixels was enhanced by employing the Excess Green Index (ExG). This modifies the hue of the image and is computed as follows
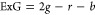
where, *r*, *g*, and *b* are the red, green, and blue color model components, respectively.^44^ Otsu’s automatic thresholding method^45^ was subsequently employed to identify optimum thresholds from the ExG image to extract greenspace vegetation pixels. Green vegetation has a high reflectance in the green band and a low reflectance in both red and blue bands. Based on this principle, greenspace was identified from the GSV images and was subsequently quantified using the greenspace metric, Green View Index (GVI).^32−36^ GVI is measured as a percentage from 0 to 100, with 100 having a maximum density of greenspace. GVI was calculated for each location using the following equation
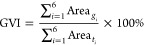
where Area_*gi*_ is the number of green pixels in the image *i*, and Area_*ti*_ is the total number of pixels in the image *i*. Li et al.^43^ tested this classification algorithm by manually classifying greenspace from a random sample of GSV images (*n* = 100). A correlation coefficient of 0.94 was observed, thereby ensuring high accuracy of the methods employed in this study. The mean GVI was calculated for each Small Area.

### 2.3 Quantifying NDVI Using Satellite Imagery and Computational Algorithms

The NDVI greenspace metric was quantified for Dublin, Cork, and Galway cities. NDVI is measured between −1 and 1, with values closer to 1 indicating more greenspace.^32,36,46−48^ NDVI is computed by applying computational algorithms to satellite imagery. NDVI is determined based on the principle that green vegetation absorbs red light and reflects near-infrared light.^46^ NDVI greenspace classification was completed using Landsat-8 satellite imagery in 30 m × 30 m grid-cells, downloaded from the U.S. Geological Survey Earth Explorer^49^ for May 2020. Landsat images were screened for cloud, shadow, and water using an Fmask filter.^50,51^ Across the three cities, 380 553 grid-cells satisfied the Fmask quality control conditions. NDVI was calculated in each grid-cell using Landsat-8 imagery as follows
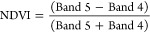
where Band 4 measures red light and Band 5 measures near-infrared light. The mean NDVI was determined for each Small Area.

### 2.4 Health, Socioeconomic, and Air Quality Variables

Health and socioeconomic measures were obtained from 2016 census data.^39^ The following variables were obtained for each Small Area: average age, percentage of females, self-reported health (number and percentage of residents whose self-evaluated health status was “good or very good”), unemployment rate, active transport mode usage rate (percentage of residents walking or cycling regularly), and third-level education attainment rate (percentage of residents aged 15 years and over with at least an ordinary bachelor’s degree). Small Areas are regions within Electoral Divisions, which contain between 80 and 120 dwellings and are used for the compilation of census statistics.^39^ The household median gross income was obtained for each Electoral Division and assigned to each Small Area within the respective Electoral Division. The 2016 annual PM_2.5_ concentrations, modeled in five zones for Dublin city using ADMS-Urban, were obtained from the Irish Environmental Protection Agency (see Figure S1).^52^ The median PM_2.5_ concentration was assigned to each Small Area located within each zone.

### 2.5 Population-Weighted Exposure to Greenspace and Statistical Analysis

Following the determination of GVI and NDVI for the three study domains, univariate outliers were removed from the data. Subsequently, population-weighted exposure to GVI and NDVI was computed for each Small Area (*pwe* to GVI and *pwe* to NDVI, respectively) according to the following equation^53^
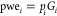
where *pwe*_*i*_ is the population-weighted exposure to GVI or NDVI in each Small Area *i; p_i_* is the population living in each Small Area *i*; and *G*_*i*_ is the GVI or NDVI greenspace in area *i*. The overall population-weighted exposure to GVI and NDVI for each city (PWE to GVI and PWE to NDVI, respectively) was calculated as follows
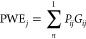
where PWE_*j*_ is the overall population-weighted exposure in each city *j*; *P*_*ij*_ is the percentage of the total population living in each Small Area *i* in each city *j*; *G*_*ij*_ has been defined previously; and *n* is the number of Small Areas in each city. Summary statistics were determined for all greenspace metrics and socioeconomic and health variables for each city utilizing the Small Area data set. Following this, the distributions of the greenspace metrics were examined. We also computed Pearson’s correlations of the greenspace metrics (GVI, NDVI, and natural log-transformed *pwe* metrics).

The lower and upper quartiles (i.e., 25th and 75th percentiles) of GVI, NDVI, *pwe* to GVI, and *pwe* to NDVI were computed for the three cities and all three cities combined. Descriptive statistics for socioeconomic and health variables were computed for Small Areas corresponding to the lower and upper greenspace quartiles. For each variable, Mann–Whitney *U*-tests were applied to assess statistically significant differences between the lower and upper quartiles. *P*-values <0.05 were considered statistically significant, and <0.001 were highly statistically significant. Statistical analyses were performed in Python version 2.7.^54^

A negative binomial regression model was employed to determine associations between greenspace exposures and counts of self-reported “good or very good” health for each Small Area. A negative binomial regression model is suitable for predicting overdispersed count data such as our self-reported “good or very good” health metric.^54−59^ We controlled for average age, percentage of females, active transport mode usage rate, third-level education attainment rate, and household median income. We used the population of each Small Area as an offset variable. These variables were selected to control for known confounding effects and were employed in similar analyses exploring health effects of greenspace.^55−59^ To reduce the skew of the distributions, the natural log was applied to average age, household median income, *pwe* to GVI, and *pwe* to NDVI. Models were run for each greenspace exposure metric (GVI, NDVI, *pwe* to GVI, and *pwe* to NDVI), for each city, and the combined city data set. Furthermore, we ran a model for an additional greenspace metric, which considered both GVI and NDVI in combination. For this, we normalized GVI and NDVI and subsequently computed a GVI/NDVI ratio for each Small Area.^32^ Sensitivity tests were performed by including PM_2.5_ concentration levels (natural log-transformed) in the regression models for the Dublin city data set.

We also conducted stratified analyses to assess potential effect modification by age, gender, and socioeconomic status. Small Areas were stratified into quartiles based on average age, percentage of females, third-level education attainment rate, and household median income. Base models were run, whereby the variable that the data were stratified by was excluded from the model, and subsequently, models were run for all Small Areas corresponding to each quartile. The regression models were performed in R version 4.0.3 using “MASS” package version 7.3.^60^

## 3 Results

### 3.1 GVI, NDVI, and Population-Weighted Exposure to Urban Greenspace

GVI was quantified for 125 274 point locations across three cities using 751 644 street-level images. NDVI was computed at a 30 m × 30 m resolution. The highest mean GVI of 20.37 (8.59) and NDVI of 0.29 (0.09) were observed in Cork city. Dublin city had both the lowest level of greenspace and the lowest rate of “good or very good” self-reported health at 83.47% (8.71%). Galway had the highest rate of third-level education attainment at 41.63% (16.17%), while Dublin had the highest median income at €50 223 (€14 123). See Table 1 for a summary of the results.

**Table 1:** Summary Statistics Including the Mean (μ) and Standard Deviation (δ) for Greenspace, Socioeconomic, and Health Variables for All Small Areas in Dublin City, Cork City, Galway City and for All Three Cities (Dublin, Cork, and Galway) Combined

	All cities	Dublin city	Cork city	Galway city
	*n* = 3335	*n* = 2179	*n* = 848	*n* = 308
	μ (δ)	μ (δ)	μ (δ)	μ (δ)
GVI (%)	15.85 (8.23)	13.86 (7.56)	20.37 (8.59)	17.46 (6.30)
NDVI	0.23 (0.09)	0.19 (0.07)	0.29 (0.09)	0.27 (0.08)
*pwe* to GVI	4074 (2743)	3515 (2491)	5328 (3011)	4579 (2418)
*pwe* to NDVI	58.24 (32.22)	49.28 (25.88)	76.43 (36.72)	71.59 (33.77)
PWE to GVI	16.16	14.01	20.97	17.91
PWE to NDVI	0.23	0.20	0.30	0.28
Population (persons)	252 (94)	251 (101)	254 (77)	256 (86)
Area (km^2^)	0.11 (0.362)	0.06 (0.20)	0.22 (0.57)	0.16 (0.39)
Age (years)	37.31 (6.04)	37.52 (5.67)	37.42 (6.56)	35.51 (6.81)
Unemployment (%)	7.10 (5.08)	7.37 (5.26)	6.43 (4.74)	7.07 (4.53)
Active transport mode usage (%)	32.25 (17.06)	35.78 (14.80)	23.70 (17.88)	30.94 (20.81)
“Good or very good” health (%)	84.41 (8.35)	83.47 (8.71)	86.20 (7.29)	86.10 (7.42)
Third-level education (%)^a^	36.65 (21.28)	37.40 (22.82)	32.93 (17.96)	41.63 (16.17)
Median income (€)	49281 (13529)	50223 (14123)	48534 (13265)	44541 (7612)

a The percentage of residents aged 15 years and over with at least an ordinary bachelor’s degree.

**Figure 2:**
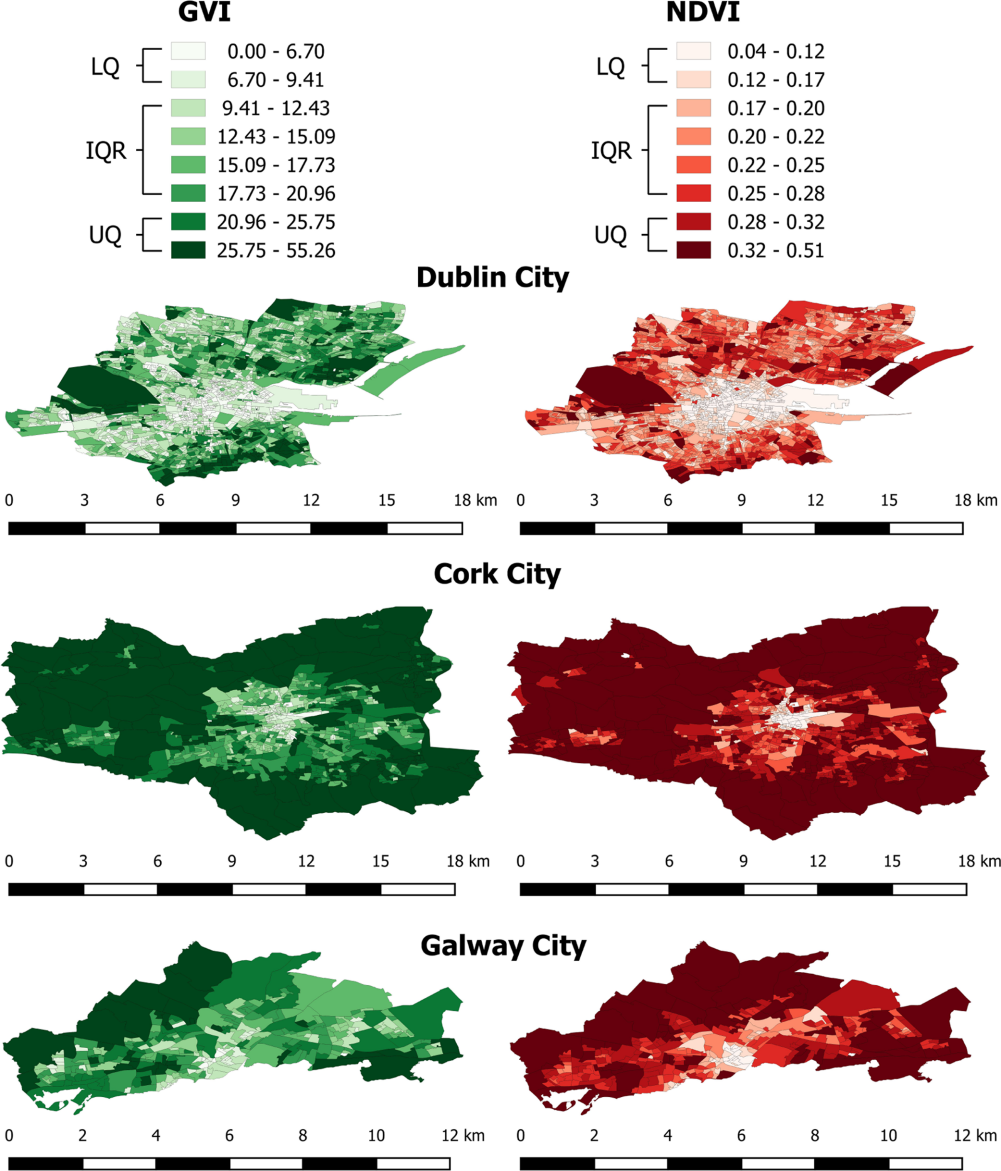
Map of the GVI and NDVI greenspace metrics per Small Area in Dublin, Cork, and Galway cities, where GVI and NDVI have been categorized as octiles. The GVI was computed using a combination of street-level GSV imagery and computer vision methods, while the NDVI was determined by processing satellite imagery using computational algorithms.

We examined the spatial differences and similarities of GVI and NDVI within the three cities (see Figures 2 and S2). While lower greenspace levels were consistently observed in urban centers, the level of greenspace varied in many Small Areas depending on the greenspace metric used. Dublin city suburbs had highly variable levels of GVI, while greenspace determined by NDVI was more consistent. Cork city suburbs were identified as areas in the top quartile of both greenspace metrics. This was similar for Galway city, while Dublin city center had a high number of Small Areas within the lower quartile of both metrics.

Population-weighted exposure to greenspace was determined in all Small Areas, and the overall PWE to greenspace was computed for each city. Cork city had the highest mean *pwe* to GVI and NDVI, while both metrics were notably lower for Dublin city. A similar trend was observed when comparing the overall PWE to GVI and NDVI (see Table 1). Figure S3 shows the *pwe* to GVI and *pwe* to NDVI mapped for each city. Changes from Figures 2 to S3 include a higher number of Small Areas in the upper quartile of *pwe* to GVI in Dublin city center. A similar trend was identified across the city for *pwe* to NDVI. To gain insight into such changes, an additional metric was developed (see the note in the Supporting Information).

The distributions of Small Area GVI, *pwe* to GVI, and *pwe* to NDVI in each city were examined and found to be right-skewed (see Figure S6). In contrast, the NDVI distributions were normally distributed. Pearson’s correlation between all greenspace metrics was positive. GVI and NDVI had a strong positive correlation of 0.71, while a correlation of 0.81 was determined for *pwe* to GVI and *pwe* to NDVI (see Table S1).

### 3.2 Comparing Socioeconomic and Health Determinants by Greenspace Quartiles

Lower and upper quartiles of GVI, NDVI, *pwe* to GVI, and *pwe* to NDVI were computed, and descriptive statistics for socioeconomic and health variables were determined within each city and for all three cities combined (see Tables 2 and S2). Residents whose self-reported health was “good or very good” were consistently higher in areas in the upper quartiles of greenspace. For the combined data set, the percentage of the population with self-reported “good or very good” health was 7% higher in the upper quartile of GVI than in the lower quartile. Notably, the population was older in the upper quartile. Lower unemployment rates and higher median gross income were observed in areas within the upper quartiles of greenspace. Third-level education attainment rates were consistently higher for people living in areas in the upper quartile of GVI. A similar trend was observed for third-level education rates for NDVI in Cork city. However, the rates were similar in the upper and lower quartiles of NDVI in Dublin and Galway cities.

**Table 2:** Summary Statistics for the Lower Quartiles (LQs) and Upper Quartiles (UQs) of GVI and NDVI Urban Greenspace Metrics^a^

	All cities				Dublin city				Cork city				Galway city			
	GVI		NDVI		GVI		NDVI		GVI		NDVI		GVI		NDVI	
	LQ	UQ	LQ	UQ	LQ	UQ	LQ	UQ	LQ	UQ	LQ	UQ	LQ	UQ	LQ	UQ
	*n* = 834	*n* = 834	*n* = 834	*n* = 834	*n* = 545	*n* = 545	*n* = 545	*n* = 545	*n* = 212	*n* = 212	*n* = 212	*n* = 212	*n* = 77	*n* = 77	*n* = 77	*n* = 77
	μ (δ)	μ (δ)	μ (δ)	μ (δ)	μ (δ)	μ (δ)	μ (δ)	μ (δ)	μ (δ)	μ (δ)	μ (δ)	μ (δ)	μ (δ)	μ (δ)	μ (δ)	μ (δ)
GVI (%)	6.30 (2.22)	27.00 (5.33)	8.39 (4.62)	23.10 (7.75)	5.26 (1.94)	24.26 (4.80)	7.25 (4.26)	20.08 (7.62)	10.31 (2.67)	31.86 (5.74)	12.85 (5.66)	28.72 (8.05)	9.53 (2.04)	25.67 (3.66)	12.14 (4.66)	21.86 (6.16)
NDVI	0.15 (0.06)	0.30 (0.08)	0.12 (0.03)	0.34 (0.05)	0.13 (0.05)	0.25 (0.05)	0.11 (0.03)	0.27 (0.03)	0.20 (0.08)	0.37 (0.07)	0.18 (0.06)	0.41 (0.04)	0.21 (0.08)	0.33 (0.77)	0.17 (0.05)	0.37 (0.05)
*pwe* to GVI	1488 (843)	7070 (2807)	1945 (1555)	6214 (2861)	1292 (835)	6166 (2829)	1733 (1553)	5230 (2690)	2344 (1039)	8767 (2860)	2936 (2046)	8014 (3193)	2126 (936)	6859 (2410)	2692 (1661)	6043 (2707)
*pwe* to NDVI	34.51 (19.94)	80.18 (35.74)	27.35 (14.94)	91.15 (32.20)	32.10 (18.57)	62.94 (29.77)	24.63 (13.13)	71.28 (25.70)	46.32 (25.93)	104.47 (37.64)	40.03 (22.61)	113.21 (34.28)	47.55 (28.03)	89.84 (37.49)	37.98 (20.34)	102.48 (33.39)
Population (persons)	235 (100)	261 (86)	231 (120)	268 (76)	241 (110)	255 (109)	236 (131)	261 (85)	223 (68)	276 (75)	222 (88)	277 (72)	221 (86)	265 (76)	216 (101)	272 (73)
Area (km^2^)	0.04 (0.14)	0.26 (0.66)	0.04 (0.14)	0.28 (0.66)	0.04 (0.17)	0.08 (0.31)	0.03 (0.17)	0.10 (0.34)	0.04 (0.08)	0.64 (1.02)	0.07 (0.15)	0.66 (1.01)	0.05 (0.05)	0.38 (0.67)	0.07 (0.09)	0.45 (0.70)
Age (years)	36.05 (5.09)	37.66 (6.01)	35.23 (5.44)	37.20 (6.74)	35.83 (4.99)	38.45 (5.57)	34.85 (5.08)	39.04 (6.10)	37.23 (6.98)	37.15 (5.75)	37.58 (7.06)	36.59 (6.13)	34.60 (6.59)	37.67 (6.87)	36.64 (6.60)	36.30 (7.36)
Unemployment (%)	8.99 (5.37)	4.72 (3.57)	8.48 (5.67)	5.78 (4.57)	9.25 (5.51)	4.92 (3.58)	8.60 (6.01)	5.61 (4.61)	9.10 (4.66)	4.14 (3.32)	8.44 (4.83)	4.82 (3.94)	8.19 (4.99)	5.45 (3.68)	6.70 (3.40)	6.42 (5.15)
Active transport mode usage (%)	42.06 (14.85)	23.54 (16.99)	47.23 (13.63)	20.09 (14.79)	43.19 (13.95)	31.61 (13.83)	49.32 (12.51)	28.19 (10.68)	37.83 (15.68)	11.91 (13.85)	39.14 (16.53)	11.63 (11.24)	39.26 (22.52)	29.39 (21.63)	49.09 (17.49)	23.63 (17.93)
“Good or very good” health (%)	80.93 (9.20)	88.00 (6.89)	80.88 (10.44)	87.62 (6.89)	80.25 (9.95)	86.74 (7.58)	79.99 (11.25)	86.08 (7.38)	81.42 (7.43)	89.45 (6.24)	81.29 (8.07)	89.39 (5.59)	84.69 (6.84)	87.92 (6.09)	84.27 (7.78)	86.76 (7.35)
Third-level education (%)^b^	33.80 (21.14)	43.98 (19.93)	40.47 (20.92)	38.08 (18.87)	33.88 (21.56)	47.81 (22.95)	41.41 (21.54)	41.79 (22.42)	27.54 (17.44)	37.60 (15.27)	29.35 (17.71)	36.16 (16.65)	40.24 (16.75)	45.94 (15.27)	41.79 (15.33)	41.69 (15.71)
Median income (€)	42 582 (10 971)	57 669 (13 315)	43 847 (12 652)	53 344 (12 389)	42 920 (10 870)	60 373 (14 410)	44 688 (12 809)	56 901 (14 606)	36 492 (8678)	57 915 (10 189)	37 627 (10 012)	56 566 (10 430)	42 086 (7306)	46 784 (8664)	39 141 (6008)	46 011 (7493)

a This includes the mean (μ) and standard deviation (δ) for greenspace metrics and socioeconomic and health variables for Dublin, Cork, and Galway cities.

b The percentage of residents aged 15 years and above with at least an ordinary bachelor’s degree.

Relative differences in socioeconomic and health variables observed between Small Areas in the lower and upper quartiles of population-weighted exposure to GVI and NDVI were examined (see Tables S3–S6). The magnitude of these differences was attenuated when examining population-weighted exposure to greenspace (*pwe* to GVI and *pwe* to NDVI). This trend was observed across most variables for all cities, with some exceptions (see Tables S3–S6). We examined differences between the distributions of the lower and upper quartiles of GVI, NDVI, *pwe* to GVI, *pwe* to NDVI, and socioeconomic and health variables. Differences were observed for almost all variables in Dublin, Cork, and Galway cities, with some minor exceptions (see Tables S3–S6).

### 3.3 Associations between Greenspace Exposure and Health

Higher greenspace exposures (GVI and NDVI) were associated with higher levels of self-reported “good or very good” health in Dublin and Cork cities, after controlling for confounding effects and offsetting for population levels. Higher rates of self-reported “good or very good” health were associated with higher GVI exposure in Galway city. For the combined city data set, an interquartile range (IQR) increase in GVI exposure was associated with a 2.78% increase in self-reported “good or very good” health [95% confidence interval (CI): 2.25–3.31]. An IQR increase in NDVI exposure was associated with a 3.51% [95% CI: 3.05–3.96] increase in self-reported “good or very good” health. An IQR increase in GVI/NDVI ratio corresponded to a 2.56% increase in self-reported “good or very good” health [95% CI: 2.01–3.11]. Associations were attenuated when examining the *pwe* metrics. An IQR increase in *pwe* to GVI and *pwe* to NDVI was associated with a 1.10% [95% CI: 0.61–1.60] and a 1.58% [95% CI: 1.08–2.08] increase in self-reported “good or very good” health, respectively (see Table 3).

**Table 3:** Difference in Counts of Self-Reported “Good or Very Good” Health Outcomes Associated with an IQR Increase in Exposure to Greenspace (GVI, NDVI, GVI/NDVI, *pwe* to GVI, and *pwe* to NDVI) as Observed for All Small Areas in Dublin City (*n* = 2179), Cork City (*n* = 848), and Galway City (*n* = 308) and All Cities (*n* = 3335)^a^^,^^b^

	Difference in counts of self-reported “good or very good” health outcome per IQR increase in exposure to greenspace (95% CI)				
City	GVI	NDVI	GVI/NDVI	*pwe* to GVI^c^	*pwe* to NDVI^c^
All cities	2.78 (2.25, 3.31)	3.51 (3.05, 3.96)	2.56 (2.01, 3.11)	1.10 (0.61, 1.60)	1.58 (1.08, 2.08)
Dublin city	1.54 (0.82, 2.25)	4.35 (3.65, 5.06)	1.39 (0.68, 2.10)	0.52 (−0.16, 1.19)	1.67 (0.96, 2.38)
Cork city	1.39 (0.57, 2.21)	1.36 (0.66, 2.06)	1.30 (0.46, 2.14)	0.02 (−0.83, 0.88)	–0.06 (−0.77, 0.65)
Galway city	2.61 (1.32, 3.90)	0.58 (−0.38, 1.54)	0.99 (0.41, 1.57)	1.14 (−0.11, 2.40)	–0.10 (−1.26, 1.07)
Dublin city^d^	1.31 (0.61, 2.02)	3.81 (3.09, 4.53)	1.18 (0.48, 1.89)	0.19 (−0.48, 0.85)	1.03 (0.32, 1.75)

a All models were adjusted for average age, percentage of females, active transport mode usage rate, third-level education attainment rate, and household median income.

b The population of each Small Area was used as an offset variable.

c Natural log-transformed.

d Sensitivity test, included an additional variable (PM_2.5_ concentration levels) in the regression model.

A sensitivity test was conducted by additionally controlling for PM_2.5_ concentration levels in Dublin City. A slight decrease (approximately 0.5%) was observed in the change in counts of self-reported “good or very good” health for an IQR increase in GVI and NDVI greenspace exposures.

The results were modified when the data were stratified by Small Areas corresponding to the quartiles of average age, percentage of females, third-level education attainment rate, and household median income relative to their base model (see Table 4 for details). Associations between greenspace metrics and self-reported “good or very good” health were strongest in the third quartile for age. The differences in counts of self-reported “good or very good” health outcomes associated with an IQR increase in greenspace exposure were highest in the Small Areas where the percentage of females and third-level education attainment rate were lowest. Increases in self-reported “good or very good” health in response to greenspace were highest in quartile 3 for income. There is evidence of effect modification.

**Table 4:** Difference in Counts of Self-Reported “Good or Very Good” Health Outcomes Associated with an IQR Increase in Exposure to Greenspace (GVI, NDVI, GVI/NDVI, *pwe* to GVI, and *pwe* to NDVI) as Observed for Stratified Quartiles for All Small Areas for the Combined City Data Set^a^^,^^b^^,^^c^

	Difference in counts of self-reported “good or very good” health outcome per IQR increase in exposure to greenspace (95% CI)				
Model	GVI	NDVI	GVI/NDVI	*pwe* to GVI^d^	*pwe* to NDVI^d^
Average Age (*n* = 3335, 834, 833, 834, 834)^e^					
Base model	2.58 (2.03, 3.14)	3.52 (3.05, 3.99)	2.31 (1.74, 2.88)	1.21 (0.70, 1.72)	1.87 (1.35, 2.38)
LQ	2.73 (1.81, 3.65)	3.13 (2.42, 3.84)	2.44 (1.52, 3.36)	1.64 (0.86, 2.42)	1.87 (1.11, 2.62)
Q2	3.21 (2.19, 4.24)	3.25 (2.37, 4.12)	2.96 (1.92, 3.99)	1.80 (0.90, 2.69)	1.99 (1.10, 2.88)
Q3	3.61 (2.70, 4.53)	4.80 (3.97, 5.63)	3.43 (2.48, 4.38)	2.36 (1.42, 3.30)	3.70 (2.68, 4.71)
UQ	1.92 (0.58, 3.27)	3.83 (2.62, 5.04)	1.65 (0.4, 3.05)	–0.68 (−2.03, 0.67)	0.33 (−1.08, 1.74)
Percentage of Females (*n* = 3335, 833, 834, 835, 833)^f^					
Base model	2.87 (2.33, 3.42)	3.73 (3.27, 4.18)	2.65 (2.10, 3.20)	1.31 (0.81, 1.81)	1.93 (1.43, 2.43)
LQ	5.06 (3.75, 6.37)	5.76 (4.76, 6.77)	4.58 (3.26, 5.90)	2.37 (1.25, 3.49)	3.58 (2.49, 4.67)
Q2	2.85 (1.81, 3.88)	3.29 (2.38, 4.20)	2.64 (1.57, 3.72)	0.68 (−0.31, 1.67)	0.84 (−0.20, 1.88)
Q3	2.05 (1.08, 3.03)	2.59 (1.79, 3.40)	1.93 (0.93, 2.93)	1.33 (0.38, 2.28)	1.66 (0.73, 2.60)
UQ	1.40 (0.47, 2.34)	2.50 (1.61, 3.39)	1.24 (0.29, 2.19)	0.30 (−0.59, 1.19)	0.41 (−0.52, 1.34)
Third-level Education Attainment Rate (*n* = 3335, 834, 833, 834, 834)^g^					
Base model	2.81 (2.27, 3.35)	3.23 (2.77, 3.70)	2.65 (2.10, 3.20)	0.87 (0.37, 1.37)	0.99 (0.49, 1.49)
LQ	3.70 (2.50, 4.90)	5.21 (4.17, 6.26)	3.46 (2.22, 4.70)	1.79 (0.68, 2.90)	2.77 (1.58, 3.96)
Q2	3.59 (2.35, 4.82)	4.40 (3.35, 5.45)	3.60 (2.32, 4.88)	1.31 (0.12, 2.51)	1.64 (0.40, 2.87)
Q3	2.70 (1.71, 3.70)	2.99 (2.23, 3.75)	2.35 (1.34, 3.37)	1.14 (0.27, 2.00)	1.76 (0.93, 2.59)
UQ	0.97 (0.20, 1.75)	0.97 (0.24, 1.71)	0.85 (0.08, 1.62)	0.22 (−0.50, 0.93)	0.20 (−0.52, 0.92)
Household Median Income (*n* = 3335, 831, 829, 872, 803)^h^					
Base model	3.89 (3.39, 4.39)	4.24 (3.80, 4.68)	3.72 (3.21, 4.23)	2.30 (1.83, 2.77)	2.58 (2.09, 3.07)
LQ	3.08 (1.60, 4.56)	3.46 (2.24, 4.68)	2.87 (1.44, 4.30)	0.74 (−0.31, 1.79)	0.62 (−0.44, 1.67)
Q2	3.01 (1.91, 4.11)	3.65 (2.79, 4.50)	2.66 (1.55, 3.77)	1.05 (0.16, 1.95)	2.10 (1.16, 3.03)
Q3	3.58 (2.55, 4.61)	4.35 (3.48, 5.21)	3.35 (2.28, 4.42)	1.76 (0.75, 2.77)	2.48 (1.44, 3.51)
UQ	1.82 (1.00, 2.63)	2.23 (1.41, 3.06)	1.74 (0.89, 2.60)	0.87 (−0.10, 1.85)	0.84 (−0.13, 1.80)

a In addition to the stratified quartile models, base models were run for the full data set (*n* = 3335). The variable by which the data were stratified was omitted from the models.

b In each set of models, we excluded the effect modifier category variable. Models were adjusted for average age, percentage of females, active transport mode usage rate, third-level education attainment rate, and household median income.

c The population of each small area was used as an offset variable.

d Natural log-transformed.

e Average age: LQ: 21-33, Q2: 33-37, Q3: 37-41, UQ: 41-73.

f Percentage of females: LQ: 20-49%, Q2: 49–51%, Q3: 51–54%, UQ: 54–76%.

g Third-level education attainment rate: LQ: 0–18%, Q2: 18–36%, Q3: 36–53%, UQ: 53–97%.

h Household median income: LQ: €18759–€38 444, Q2: €38 444–€46 842, Q3: €46 842–€58 130, UQ: €58 130–€96 130.

## 4 Discussion

Exposure to street-level greenspace is currently unquantified in high resolution and understudied worldwide. An increased understanding of exposure to greenspace is needed to fully yield its socioeconomic and health benefits. This study sought to address this major knowledge gap. The methodology utilized in this research demonstrated a scalable and novel approach to quantifying greenspace and population-weighted exposure to greenspace within and among cities on a national scale. In this first-of-a-kind study, GVI was computed for 125 274 locations in three Irish cities using 751 644 GSV images and computer vision methods. Further analyses provided insight into how greenspace and population-weighted exposure to greenspace varied with socioeconomic factors and self-reported health.

The spatial similarities and differences of GVI and NDVI were examined, identifying low greenspace levels in city centers. GVI and NDVI were not matched in greenspace quantity in some Small Areas. Similar findings were observed by Lu et al.^61^ who compared greenspace distribution in Hong Kong. Lack of greenspace development within city centers may be due to demand for commercial space. Modern greenspaces such as skyrise greenery and green roofs must be considered in areas where competition exists for open space.

In our study, higher median income and lower unemployment levels were observed in upper quartile greenspace areas. Previous studies have reported that areas with lower household income had significantly less greenspace development.^23,32^ With urbanization continuing at a rapid rate, this threatens further socioeconomic inequities of greenspace accessibility. It is important that future greenspace development is distributed among societies to eliminate inequalities.

We observed that the percentage of people who use active transport modes was higher in the lower quartiles of greenspace than the upper quartiles. Residents in city centers, which account for a significant number of Small Areas in the lower quartiles, are more likely to actively commute as their journeys are shorter. With that in mind, greenspace development is vital in city centers and peripheries as commuters are beneficiaries of greenspace. Studies have shown that greenspace alleviates environmental issues such as air pollution^17^ while also acting as a natural barrier against such emissions, reducing exposure to those who travel adjacent to them. Commuters’ journeys could be enhanced and their health improved as a result of higher greenspace exposure.

A directed acyclic graph (DAG) was used to identify confounders, mediators, and effect modifiers (see Figure S7).^55−59,62,63^ While controlling for identified factors, an interquartile range increase in street-level greenspace was associated with a 2.78% increase in counts of self-reported “good or very good” health [95% CI: 2.33–3.42]. We also found evidence of effect modification by age, gender, and socioeconomic status in associations between counts of self-reported “good or very good” health with greenspace exposure. Such associations identified between greenspace and self-reported health were determined at the Small Area level. Although this is the smallest unit of aggregated data used for the compilation of census statistics, we cannot exclude the possibility of ecological fallacy.^64^ Therefore, we are unable to assume that such associations exist at individual level.

Previous studies have compared traditional greenspace metrics, such as NDVI, with self-reported health.^47,55,56,65−67^ Associations between greenspace exposure and self-reported health that we identified were consistent with such studies, with better health outcomes observed for residents in greener areas.^10,55,58,59^ However, the impact of greenspace exposure on self-reported health may differ by greenspace metric, including our GVI exposure metric. Therefore, we cannot directly compare our results with the previous literature.

General health is investigated in the Irish census based on participants’ response to a single qualitative question: “how is your health in general?” Although it is a subjective assessment of health, studies have found that self-reported health is a strong predictor of objective health measures and mortality.^68−72^ However, other studies determined that self-reported health underestimates the magnitude of health inequalities by socioeconomic status.^73,74^ Under Irish legislation, it is compulsory for everyone in Ireland to complete or be included on a census form.^75^ Ninety-nine percent of the population were accounted for by completed census forms, while basic demographic information was included for the remaining 1% to ensure completeness of the data.^76^

By using novel computer vision methods to identify greenspace, this research has made significant progress in examining urban environmental engineering problems using computer science-derived methodologies. Although computationally intensive, automatic processing of GSV images is cost- and time-efficient. Moreover, GSV images are obtained virtually, supporting research safety by eliminating physical surveying of potentially dangerous areas. Additionally, it is an unobtrusive method of data collection. Most GSV images are captured by a panoramic camera, which is mounted onto a car.^77^ Google have also developed a Street View Three-Wheeler to gather imagery in cities with narrow streets and a Street View Trekker, which is a wearable backpack that captures images in locations only accessible by foot.^37^ Previously, GSV images were freely available. However, Google introduced a pricing plan for GSV API of $0.0056 per image. The total cost for this study is approximately $4200.^78^ Additionally, GSV API does not allow historical images to be collected. Therefore, green months were specified to eliminate seasonality effects.

This research assumes that greenspace exposure is determined based on its availability, which is a key requirement for greenspace use. However, other determinants contribute to greenspace use,^79^ for instance, accessibility to greenspace. Another factor that impacts greenspace use is perceived safety and security. High crime rates and antisocial behavior discourage residents from utilizing such spaces. For this large-scale study, data to provide insights for such factors are not readily available or easy to gather.

Many opportunities exist to balance urban environmental and health problems with positive access and exposure to greenspace. The cutting-edge approach adopted in this research identifies a scalable approach to benchmarking street-level and overhead greenspace in high resolution. Examining associations between higher levels of greenspace exposure and self-reported “good or very good” health, we observed positive associations in most cases. While urbanization continues at a rapid rate, the development of greenspace will become increasingly contested. The socioeconomic and health benefits of greenspace observed in this study must be recognized. Safe and accessible greenspace development must be embedded in national and international policies and become part of our global vision. This will help society develop healthy, sustainable, and livable cities of the future.

## Supporting Information Available

PM_2.5_ concentration level maps; greenspace metric comparison maps; *pwe* to GVI and *pwe* to NDVI maps; maps of GVI and NDVI, population, octiles, and *pwe* to GVI and NDVI; distribution plots; correlation matrix; summary statistics for the lower and upper quartiles of *pwe* to GVI and NDVI; tables of relative differences and the statistical significance of differences in greenspace, socioeconomic and health variables between the lower and upper quartiles of greenspace metrics; and directed acyclic graph (PDF)
