# Supplementary material for: “Biophilic Cities”: Quantifying the Impact of Google Street View-Derived Greenspace Exposures on Socioeconomic Factors and Self-Reported Health
Source: Environ Sci Technol. 2021 Jun 23;55(13):9063–73. doi: 10.1021/acs.est.1c01326 (PMC8277136; doi:10.1021/acs.est.1c01326)
Supplement: Supplementary file 1 — es1c01326_si_001.pdf [file es1c01326_si_001.pdf]

Supporting Information for the Article:

"Biophilic Cities": Quantifying the Impact of Google Street View-Derived Greenspace Exposures on Socioeconomic Factors and Self-Reported Health

Anna C. O'Regan[a,b], Ruth F. Hunter[c], Marguerite M. Nyhan[a,b,d]*

[a]*Discipline of Civil, Structural & Environmental Engineering, School of Engineering & Architecture, University College Cork, Cork, Ireland.*

[b]*MaREI Centre for Energy, Climate & Marine and Environmental Research Institute, University College Cork, Cork, Ireland.*

[c]*Centre for Public Health, Queen's University Belfast, Belfast BT12 6BA, Northern Ireland, United Kingdom.*

[d]*Harvard T.H. Chan School of Public Health, Harvard University, Boston, Massachusetts 02215, United States.*

**Corresponding Author: Tel +353214902965. Email: 116370613@umail.ucc.ie.*

Pages: 15

Figures: 7

Tables: 6

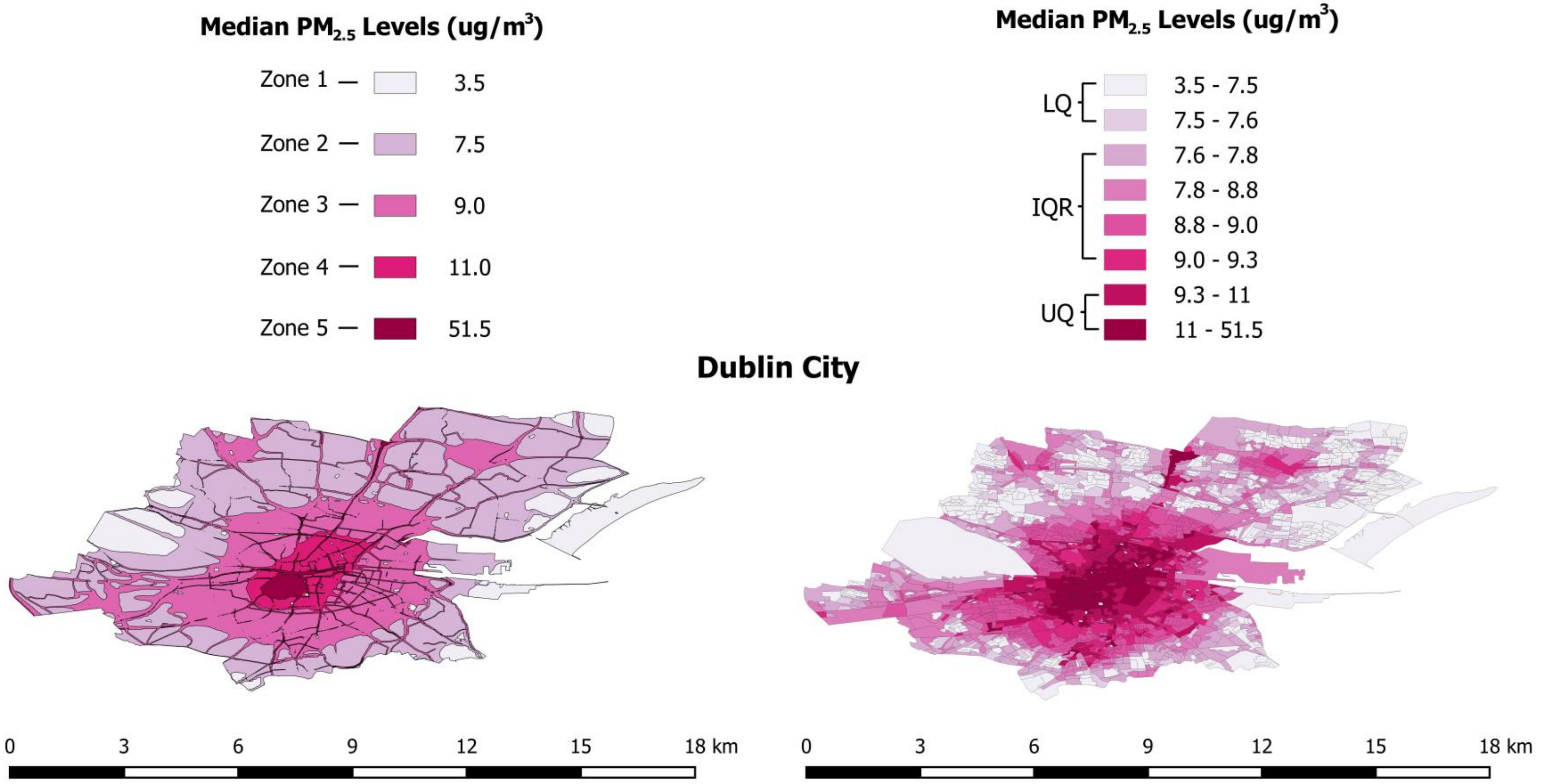

Figure S1. Map of (a) $PM_{2.5}$ concentration levels in five zones for Dublin city using the atmospheric dispersion model system ADMS-Urban (b) $PM_{2.5}$ concentration levels in each Small Area for Dublin city.

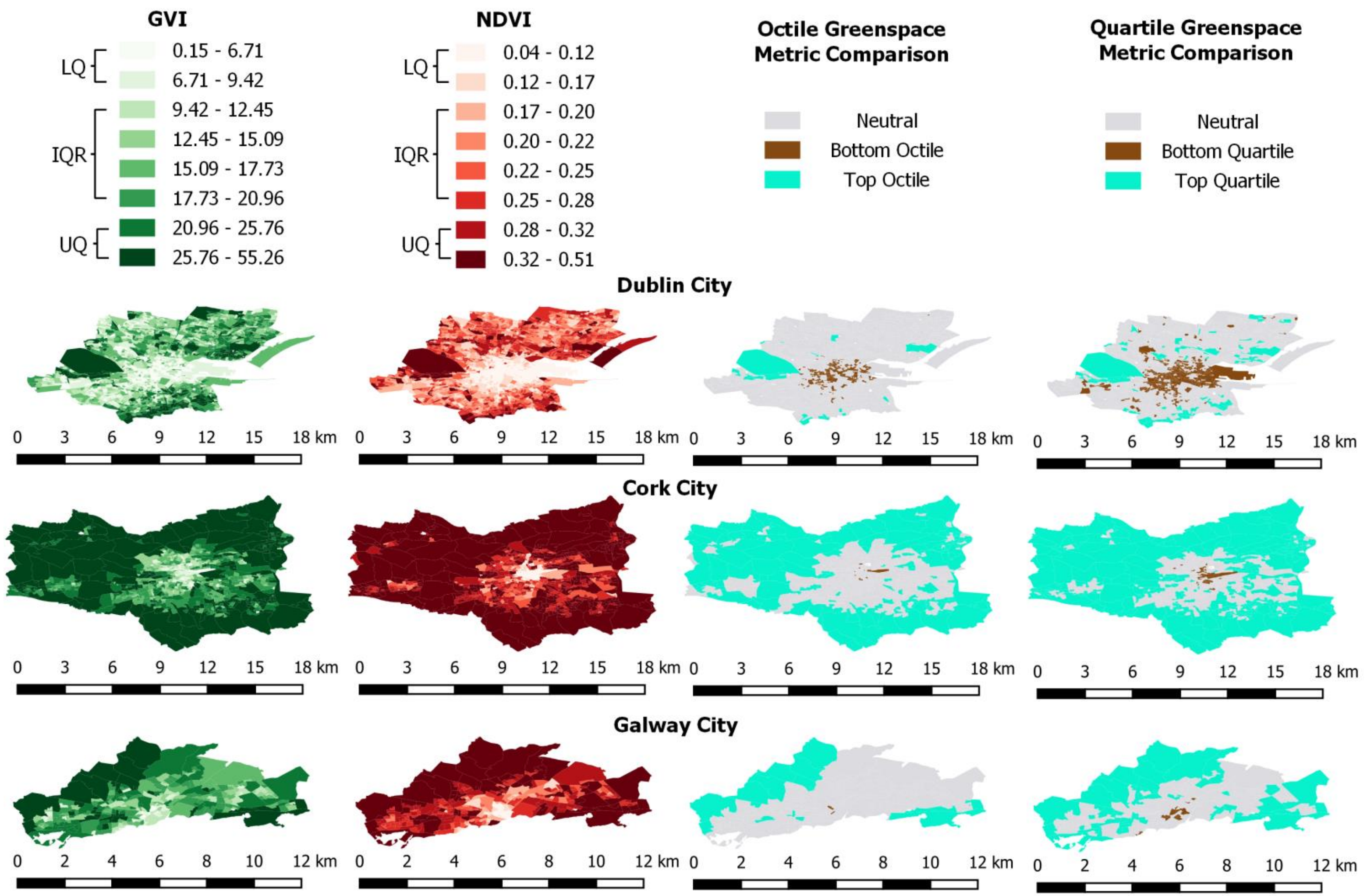

Figure S2. Map of (a) GVI (b) NDVI (c) GVI and NDVI octile comparison, where Small Areas in the highest octile (*n=204*) and lowest octile (*n=243*) of both greenspace metrics are identified (d) GVI and NDVI quartile comparison, where Small Areas in the highest quartile (*n=476*) and lowest quartile (*n=554*) of both greenspace metrics are identified.

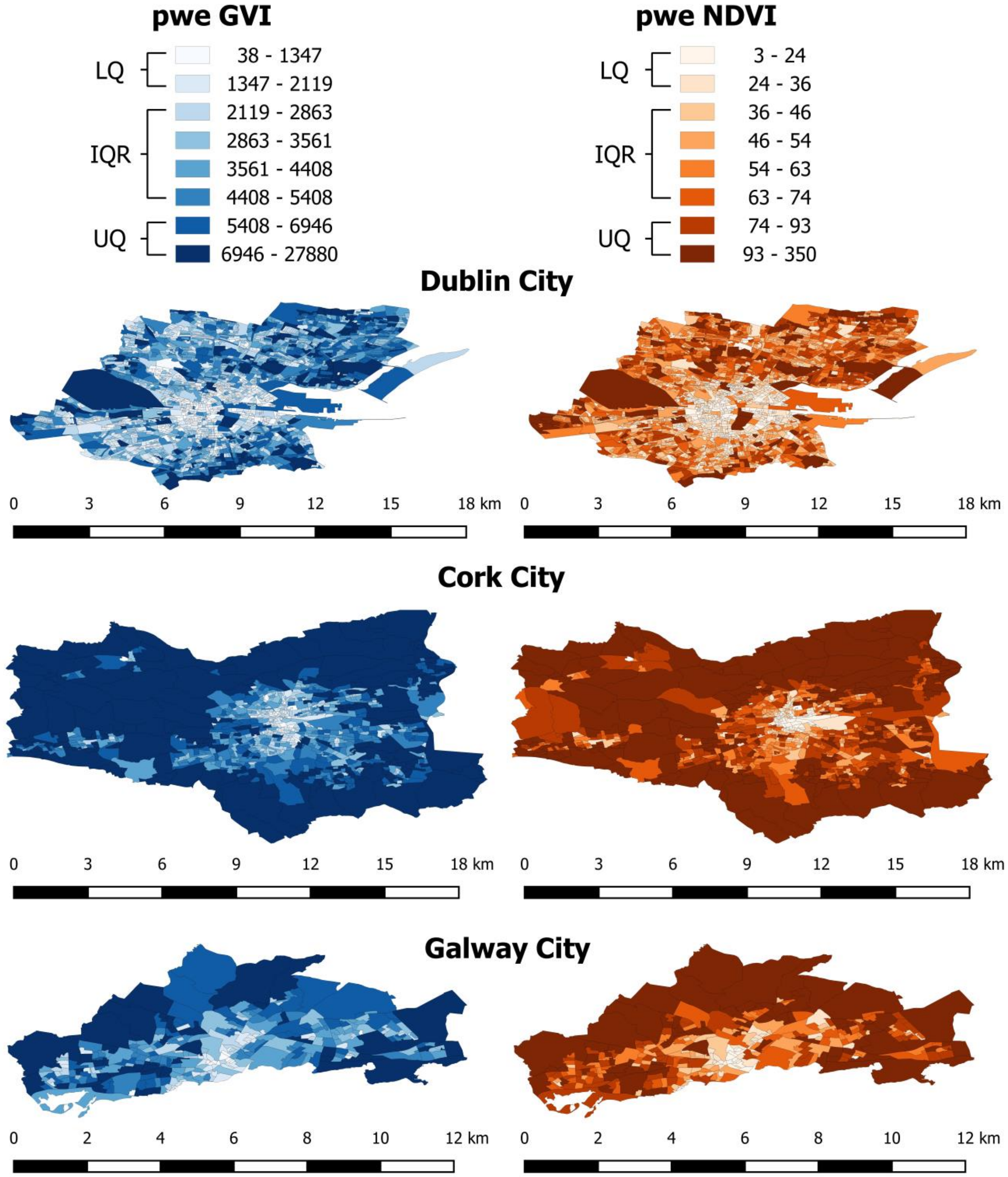

Figure S3. Map of the population-weighted exposure to GVI and NDVI greenspace metrics per Small Area in Dublin city, Cork city and Galway city. The GVI was computed using a combination of street-level GSV imagery and computer vision methods while the NDVI was determined by processing satellite imagery using computational algorithms.

Population-Weighted Exposure to Urban Greenspace

To gain insight into changes from the greenspace metrics and *pwe* metrics, an additional metric was developed whereby numbered population octiles were subtracted from numbered greenspace metric octiles, resulting in a range from -7 to +7, where -7 indicates high population and low greenspace and +7 indicates high greenspace and low population (see Figure S4 and S5). Figure S4 shows high population in Dublin city center and high level of greenspace in the south of the city contribute to high *pwe* to GVI. Figure S5 indicates a high *pwe* to NDVI in Dublin is due to high population. The west and east of Cork city have high greenspace levels and low population, while Galway city has higher greenspace in the west with a higher population in the east.

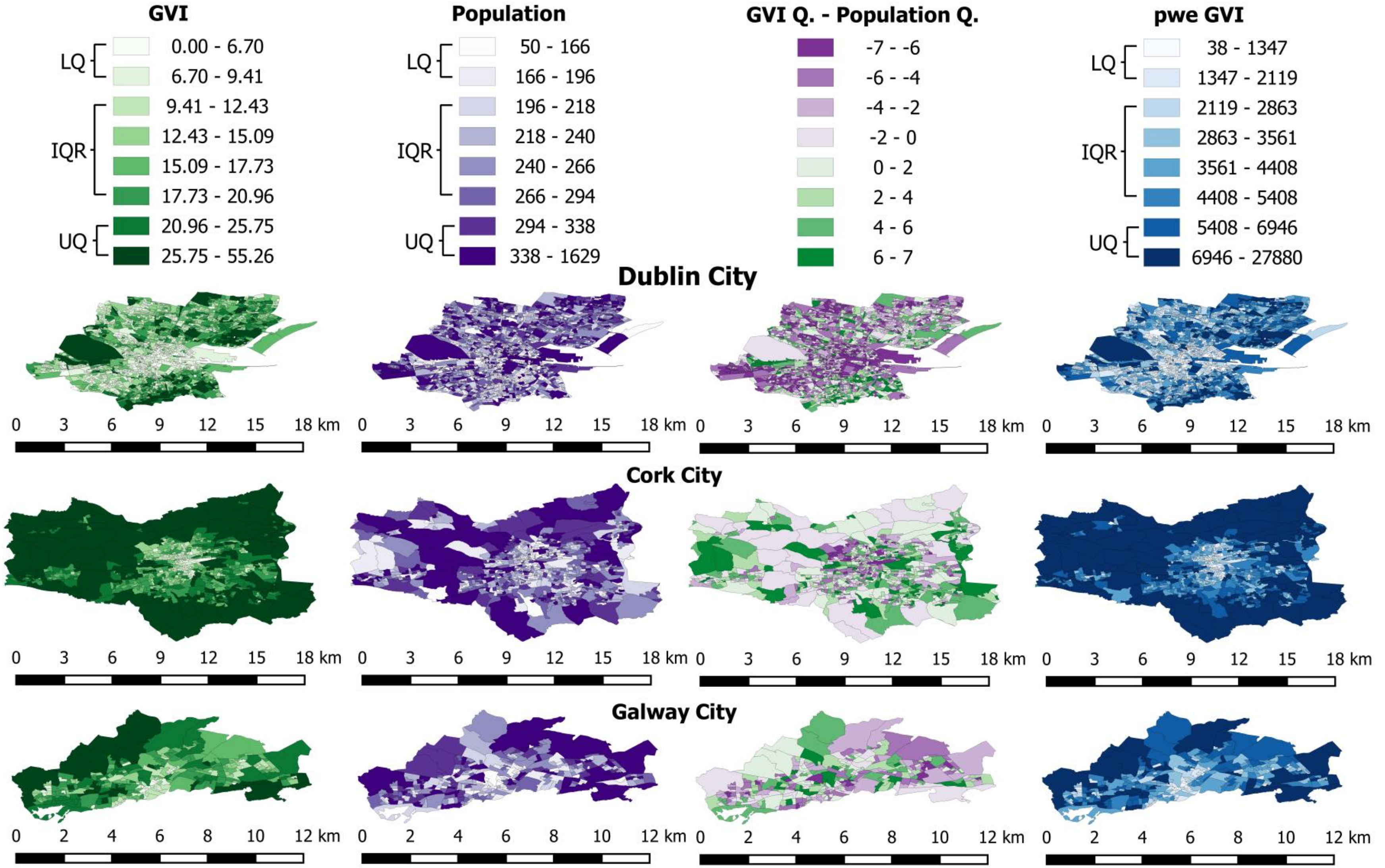

Figure S4. Map of (a) GVI (b) population (c) GVI octile – population octile, where -7 indicates an area that falls in the highest population octile and lowest GVI octile while +7 indicates an area that falls in the highest GVI octile and lowest population quartile (d) the population-weighted exposure to GVI.

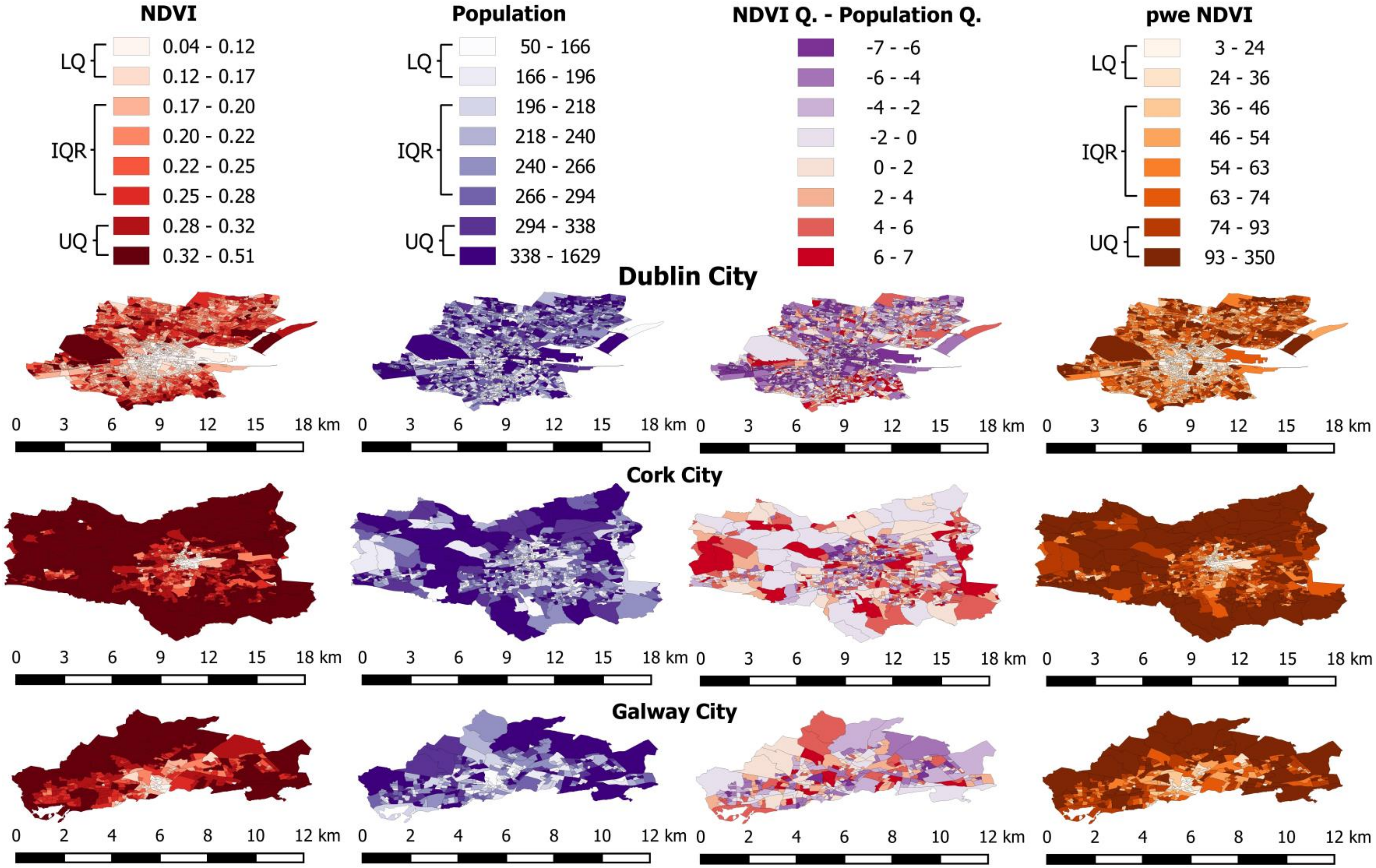

Figure S5. Map of (a) NDVI (b) population (c) NDVI octile – population octile, where -7 indicates an area that falls in the highest population octile and lowest NDVI octile while +7 indicates an area that falls in the highest NDVI octile and lowest population octile (d) the population-weighted exposure to NDVI.

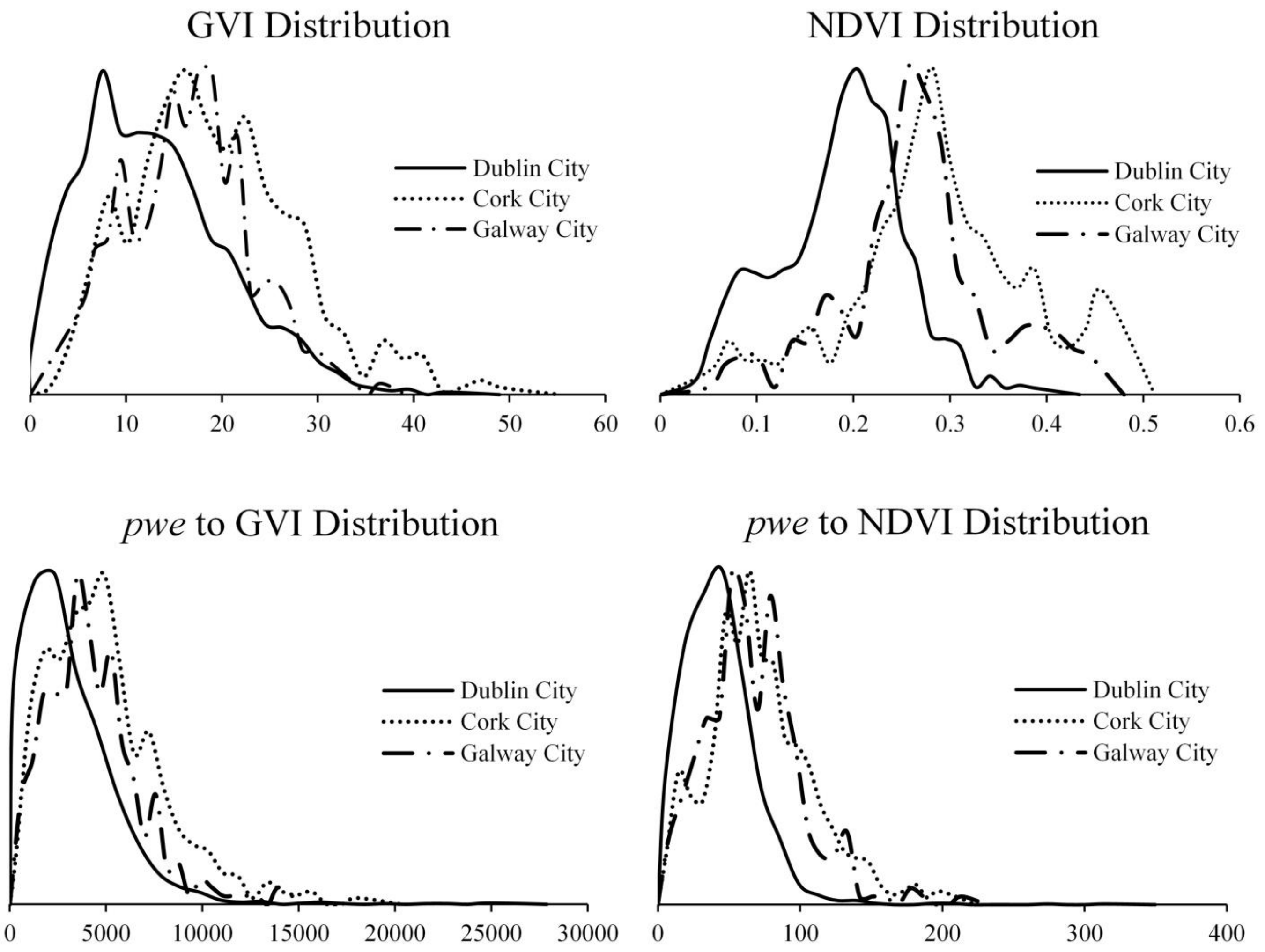

Figure S6. Plot of the distributions of GVI (top-left), NDVI (top-right), *pwe* to GVI (lower-left) and *pwe* to NDVI (lower-right) computed in each Small Area in Dublin city (*n=2179*), Cork city *(n=848)* and Galway city *(n=308)*.

Table S1. Pearson correlation of urban greenspace metrics (GVI, NDVI, natural log transformed *pwe* to GVI and natural log transformed *pwe* to NDVI) for all Small Areas in Dublin city, Cork city, Galway city and for all three cities (Dublin, Cork and Galway) combined.

	**All cities**			**Dublin city**			**Cork city**			**Galway city**		
	GVI	NDVI	*pwe* to GVI	GVI	NDVI	*pwe* to GVI	GVI	NDVI	*pwe* to GVI	GVI	NDVI	*pwe* to GVI
GVI	1.0000	-	-	1.0000	-	-	1.0000	-	-	1.0000	-	-
NDVI	0.7119	1.0000	-	0.6499	1.0000	-	0.7245	1.0000	-	0.6102	1.0000	-
pwe to GVI	0.8247	0.6678	1.0000	0.8104	0.6264	1.0000	0.8341	0.7237	1.0000	0.8074	0.6014	1.0000
pwe to NDVI	0.5709	0.8121	0.8129	0.4868	0.7740	0.7776	0.5852	0.8445	0.8513	0.5267	0.7956	0.8362

Table S2. Summary statistics for the lower and upper quartiles of *pwe* to GVI and *pwe* to NDVI. This includes the mean and standard deviation for greenspace metrics and socioeconomic and health variables for Dublin city, Cork city and Galway city.

	**All cities**				**Dublin city**				**Cork city**				**Galway city**			
	***pwe* to GVI**		***pwe* to NDVI**		***pwe* to GVI**		***pwe* to NDVI**		***pwe* to GVI**		***pwe* to NDVI**		***pwe* to GVI**		***pwe* to NDVI**	
	**LQ**	**UQ**	**LQ**	**UQ**	**LQ**	**UQ**	**LQ**	**UQ**	**LQ**	**UQ**	**LQ**	**UQ**	**LQ**	**UQ**	**LQ**	**UQ**
	n = 834	*n* = 834	*n* = 834	*n* = 834	*n* = 545	*n* = 545	*n* = 545	*n* = 545	*n* = 212	*n* = 212	*n* = 212	*n* = 212	*n* = 77	*n* = 77	*n* = 77	*n* = 77
	μ (δ)	μ (δ)	μ (δ)	μ (δ)	μ (δ)	μ (δ)	μ (δ)	μ (δ)	μ (δ)	μ (δ)	μ (δ)	μ (δ)	μ (δ)	μ (δ)	μ (δ)	μ (δ)
pwe to GVI	1298 (508)	7726 (2584)	1697 (1007)	7022 (3078)	1087 (437)	6763 (2581)	1428 (850)	6008 (3055)	2080 (669)	9458 (2404)	2449 (1170)	8750 (3031)	1892 (631)	7777 (2024)	2227 (1039)	7139 (2576)
pwe to NDVI	28.90 (14.50)	92.64 (33.93)	23.50 (7.66)	101.02 (28.34)	26.62 (13.46)	74.68 (28.14)	21.08 (6.56)	81.65 (24.95)	37.98 (16.71)	118.09 (32.30)	34.21 (13.01)	125.89 (25.60)	38.22 (18.57)	108.32 (31.29)	36.65 (6.62)	34.90 (6.89)
GVI (%)	6.96 (3.17)	25.16 (6.87)	9.62 (6.11)	21.62 (7.84)	5.89 (2.85)	22.44 (6.26)	8.30 (5.54)	18.14 (7.23)	11.27 (4.02)	30.14 (7.02)	13.44 (6.45)	26.27 (8.37)	10.33 (3.03)	23.54 (5.04)	12.68 (5.33)	21.18 (6.25)
NDVI	0.15 (0.06)	0.30 (0.08)	0.13 (0.05)	0.31 (0.07)	0.14 (0.05)	0.22 (0.05)	0.12 (0.05)	0.25 (0.05)	0.20 (0.08)	0.37 (0.07)	0.19 (0.07)	0.38 (0.06)	0.20 (0.07)	0.33 (0.77)	0.19 (0.07)	0.35 (0.07)
Population (persons)	199 (65)	321 (121)	189 (67)	331 (108)	198 (65)	318 (138)	187 (68)	337 (127)	190 (46)	322 (81)	187 (48)	337 (72)	186 (54)	338 (90)	176 (46)	341 (90)
Area (km^2)	0.03 (0.03)	0.28 (0.67)	0.03 (0.05)	0.28 (0.65)	0.02 (0.03)	0.11 (0.38)	0.02 (0.03)	0.11 (0.36)	0.03 (0.03)	0.64 (1.02)	0.05 (0.08)	0.57 (0.97)	0.04 (0.04)	0.41 (0.71)	0.05 (0.07)	0.42 (0.70)
Age (years)	36.66 (6.05)	37.26 (6.40)	35.88 (6.04)	36.75 (6.74)	36.32 (5.78)	38.07 (5.96)	35.42 (5.80)	37.90 (6.00)	37.44 (6.68)	36.34 (6.07)	37.59 (6.90)	34.75 (6.00)	35.24 (6.41)	35.99 (6.98)	36.65 (6.62)	34.90 (6.89)
Unemployment (%)	8.71 (5.29)	5.32 (4.37)	8.12 (5.57)	6.28 (5.12)	8.78 (5.43)	5.87 (4.85)	8.26 (5.81)	6.92 (5.36)	8.72 (4.73)	4.28 (3.69)	8.45 (4.68)	5.34 (4.71)	7.64 (3.88)	6.30 (4.51)	7.17 (4.02)	7.21 (5.32)
Active transport mode usage (%)	43.57 (14.84)	21.89 (15.51)	47.75 (13.54)	20.78 (14.38)	44.33 (14.29)	29.03 (12.23)	49.61 (12.34)	27.38 (10.12)	38.10 (15.69)	11.48 (13.55)	38.27 (15.60)	11.92 (12.66)	41.99 (22.36)	23.60 (18.79)	47.03 (19.66)	22.00 (17.76)
"Good or very good" health (%)	81.15 (9.05)	86.97 (8.37)	81.50 (10.09)	86.68 (8.14)	81.03 (9.30)	85.39 (9.11)	81.16 (10.50)	84.67 (8.80)	82.27 (6.85)	89.33 (6.38)	81.65 (8.30)	89.18 (6.69)	84.63 (7.44)	87.87 (7.07)	84.81 (7.43)	87.30 (7.31)
Third-level education (%) [1]	36.209 (21.35)	38.74 (19.66)	43.20 (21.00)	34.83 (18.88)	36.69 (22.20)	40.13 (23.23)	44.27 (21.87)	33.29 (21.71)	30.86 (17.66)	36.68 (15.22)	31.80 (17.481)	34.21 (16.91)	42.49 (16.04)	43.82 (15.68)	43.70 (15.18)	40.07 (15.79)
Median income (€)	43030 (11617)	55958 (12987)	45132 (13552)	52948 (12391)	44133 (11576)	57166 (14538)	46207 (13704)	53295 (14161)	37354 (10288)	57479 (10178)	38192 (10808)	56110 (9997)	41458 (8028)	46991 (8247)	40346 (7293)	46382 (7680)

[1] the percentage of residents aged 15 years and over with at least an ordinary bachelor's degree

Table S3. Relative difference between lower and upper quartiles for GVI, *pwe* to GVI, NDVI and *pwe* to NDVI for all cities. Mann-Whitney U test results for all cities.

All cities (Dublin city, Cork city and Galway city)								
	**Relative Difference** *(Upper Quartile – Lower Quartile)*				**Mann-Whitney U Test Results**			
	**GVI**	***pwe* to GVI**	**NDVI**	***pwe* to NDVI**	**GVI**	***pwe* to GVI**	**NDVI**	***pwe* to NDVI**
GVI (%)	20.70	18.20	14.71	12.01	$p < 0.001$	$p < 0.001$	$p < 0.001$	$p < 0.001$
NDVI	0.16	0.15	0.22	0.18	$p < 0.001$	$p < 0.001$	$p < 0.001$	$p < 0.001$
pwe to GVI	5581.83	6427.47	4268.77	5325.35	$p < 0.001$	$p < 0.001$	$p < 0.001$	$p < 0.001$
pwe to NDVI	45.67	63.73	63.80	77.52	$p < 0.001$	$p < 0.001$	$p < 0.001$	$p < 0.001$
Population (persons)	26	122	37	142	$p < 0.001$	$p < 0.001$	$p < 0.001$	$p < 0.001$
Area (km^2)	0.23	0.26	0.25	0.25	$p < 0.001$	$p < 0.001$	$p < 0.001$	$p < 0.001$
Age (years)	1.61	0.60	1.97	0.87	$p < 0.001$	$p < 0.05$	$p < 0.001$	$p < 0.001$
Unemployment (%)	-4.28	-3.39	-2.70	-1.84	$p < 0.001$	$p < 0.001$	$p < 0.001$	$p < 0.001$
Active transport mode usage (%)	-18.52	-21.68	-27.17	-26.98	$p < 0.001$	$p < 0.001$	$p < 0.001$	$p < 0.001$
"Good or very good" health (%)	7.07	5.82	6.75	5.19	$p < 0.001$	$p < 0.001$	$p < 0.001$	$p < 0.001$
Third-level education (%) [1]	10.18	2.65	-2.39	-8.37	$p < 0.001$	$p < 0.05$	$p < 0.05$	$p < 0.001$
Median income (€)	15087	12928	9496	7817	$p < 0.001$	$p < 0.001$	$p < 0.001$	$p < 0.001$

[1] the percentage of residents aged 15 years and over with at least an ordinary bachelor's degree

Table S4. Relative difference between lower and upper quartiles for GVI, *pwe* to GVI, NDVI and *pwe* to NDVI for Dublin city. Mann-Whitney U test results for Dublin City.

Dublin city								
	**Relative Difference** *(Upper Quartile – Lower Quartile)*				**Mann-Whitney U Test Results**			
	**GVI**	***pwe* to GVI**	**NDVI**	***pwe* to NDVI**	**GVI**	***pwe* to GVI**	**NDVI**	***pwe* to NDVI**
GVI (%)	19.00	16.55	12.84	9.83	$p < 0.001$	$p < 0.001$	$p < 0.001$	$p < 0.001$
NDVI	0.11	0.08	0.17	0.13	$p < 0.001$	$p < 0.001$	$p < 0.001$	$p < 0.001$
pwe to GVI	4874.73	5676.50	3514.41	4579.94	$p < 0.001$	$p < 0.001$	$p < 0.001$	$p < 0.001$
pwe to NDVI	30.85	48.06	46.70	60.57	$p < 0.001$	$p < 0.001$	$p < 0.001$	$p < 0.001$
Population (persons)	13	120	25	150	$p < 0.001$	$p < 0.001$	$p < 0.001$	$p < 0.001$
Area (km^2)	0.04	0.09	0.07	0.09	$p < 0.001$	$p < 0.001$	$p < 0.001$	$p < 0.001$
Age (years)	2.61	1.75	4.19	2.48	$p < 0.001$	$p < 0.001$	$p < 0.001$	$p < 0.001$
Unemployment (%)	-4.33	-2.91	-2.98	-1.35	$p < 0.001$	$p < 0.001$	$p < 0.001$	$p < 0.001$
Active transport mode usage (%)	-11.59	-15.30	-21.13	-22.23	$p < 0.001$	$p < 0.001$	$p < 0.001$	$p < 0.001$
"Good or very good" health (%)	6.49	4.36	6.09	3.52	$p < 0.001$	$p < 0.001$	$p < 0.001$	$p < 0.001$
Third-level education (%) [1]	13.92	3.44	0.38	-10.97	$p < 0.001$	$p < 0.05$	$p = 0.38$	$p < 0.001$
Median income (€)	17453	13033	12268.04	7088.26	$p < 0.001$	$p < 0.001$	$p < 0.001$	$p < 0.001$

[1] the percentage of residents aged 15 years and over with at least an ordinary bachelor's degree

Table S5. Relative difference between lower and upper quartiles for GVI, *pwe* to GVI, NDVI and *pwe* to NDVI for Cork city. Mann-Whitney U test results for Cork City.

Cork city								
	**Relative Difference** *(Upper Quartile – Lower Quartile)*				**Mann-Whitney U Test Results**			
	**GVI**	***pwe* to GVI**	**NDVI**	***pwe* to NDVI**	**GVI**	***pwe* to GVI**	**NDVI**	***pwe* to NDVI**
GVI (%)	21.55	18.87	15.88	12.83	$p < 0.001$	$p < 0.001$	$p < 0.001$	$p < 0.001$
NDVI	0.17	0.17	0.23	0.19	$p < 0.001$	$p < 0.001$	$p < 0.001$	$p < 0.001$
pwe to GVI	6423.23	7377.56	5078.23	6301.53	$p < 0.001$	$p < 0.001$	$p < 0.001$	$p < 0.001$
pwe to NDVI	58.14	80.12	73.18	91.68	$p < 0.001$	$p < 0.001$	$p < 0.001$	$p < 0.001$
Population (persons)	52	132	55	151	$p < 0.001$	$p < 0.001$	$p < 0.001$	$p < 0.001$
Area (km^2)	0.61	0.61	0.59	0.52	$p < 0.001$	$p < 0.001$	$p < 0.001$	$p < 0.001$
Age (years)	-0.07	-1.10	-1.00	-2.84	$p = 0.32$	$p = 0.08$	$p = 0.12$	$p < 0.001$
Unemployment (%)	-4.95	-4.44	-3.62	-3.11	$p < 0.001$	$p < 0.001$	$p < 0.001$	$p < 0.001$
Active transport mode usage (%)	-25.92	-26.61	-27.51	-26.34	$p < 0.001$	$p < 0.001$	$p < 0.001$	$p < 0.001$
"Good or very good" health (%)	8.02	7.06	8.10	7.53	$p < 0.001$	$p < 0.001$	$p < 0.001$	$p < 0.001$
Third-level education (%) [1]	10.06	5.82	6.82	2.41	$p < 0.001$	$p < 0.001$	$p < 0.001$	$p < 0.001$
Median income (€)	21423	20125	18939	17918	$p < 0.001$	$p < 0.001$	$p < 0.001$	$p < 0.001$

[1] the percentage of residents aged 15 years and over with at least an ordinary bachelor's degree

Table S6. Relative difference between lower and upper quartiles for GVI, *pwe* to GVI, NDVI and *pwe* to NDVI for Galway City. Mann-Whitney U test results for Galway city.

Galway city								
	**Relative Difference** *(Upper Quartile – Lower Quartile)*				**Mann-Whitney U Test Results**			
	**GVI**	***pwe* to GVI**	**NDVI**	***pwe* to NDVI**	**GVI**	***pwe* to GVI**	**NDVI**	***pwe* to NDVI**
GVI (%)	16.14	13.21	9.71	8.51	$p < 0.001$	$p < 0.001$	$p < 0.001$	$p < 0.001$
NDVI	0.13	0.12	0.20	0.16	$p < 0.001$	$p < 0.001$	$p < 0.001$	$p < 0.001$
pwe to GVI	4733.08	5884.22	3350.90	4911.79	$p < 0.001$	$p < 0.001$	$p < 0.001$	$p < 0.001$
pwe to NDVI	42.30	70.11	64.50	83.11	$p < 0.001$	$p < 0.001$	$p < 0.001$	$p < 0.001$
Population (persons)	44	151	56	165	$p < 0.001$	$p < 0.001$	$p < 0.001$	$p < 0.001$
Area (km^2)	0.32	0.36	0.38	0.37	$p < 0.001$	$p < 0.001$	$p < 0.001$	$p < 0.001$
Age (years)	3.07	0.75	-0.34	-1.75	$p < 0.001$	$p = 0.19$	$p = 0.35$	$p = 0.53$
Unemployment (%)	-2.74	-1.34	-0.27	0.04	$p < 0.001$	$p < 0.001$	$p = 0.052$	$p = 0.18$
Active transport mode usage (%)	-9.87	-18.39	-25.46	-25.03	$p < 0.05$	$p < 0.001$	$p < 0.05$	$p < 0.001$
“Good or very good” health (%)	3.24	3.24	2.50	2.49	$p < 0.001$	$p < 0.001$	$p < 0.001$	$p < 0.05$
Third-level education (%) [1]	5.70	1.34	-0.09	-3.63	$p < 0.05$	$p = 0.28$	$p = 0.41$	$p = 0.07$
Median income (€)	4697.34	5533.09	6869.95	6036.06	$p < 0.001$	$p < 0.001$	$p < 0.001$	$p < 0.001$

[1] the percentage of residents aged 15 years and over with at least an ordinary bachelor’s degree

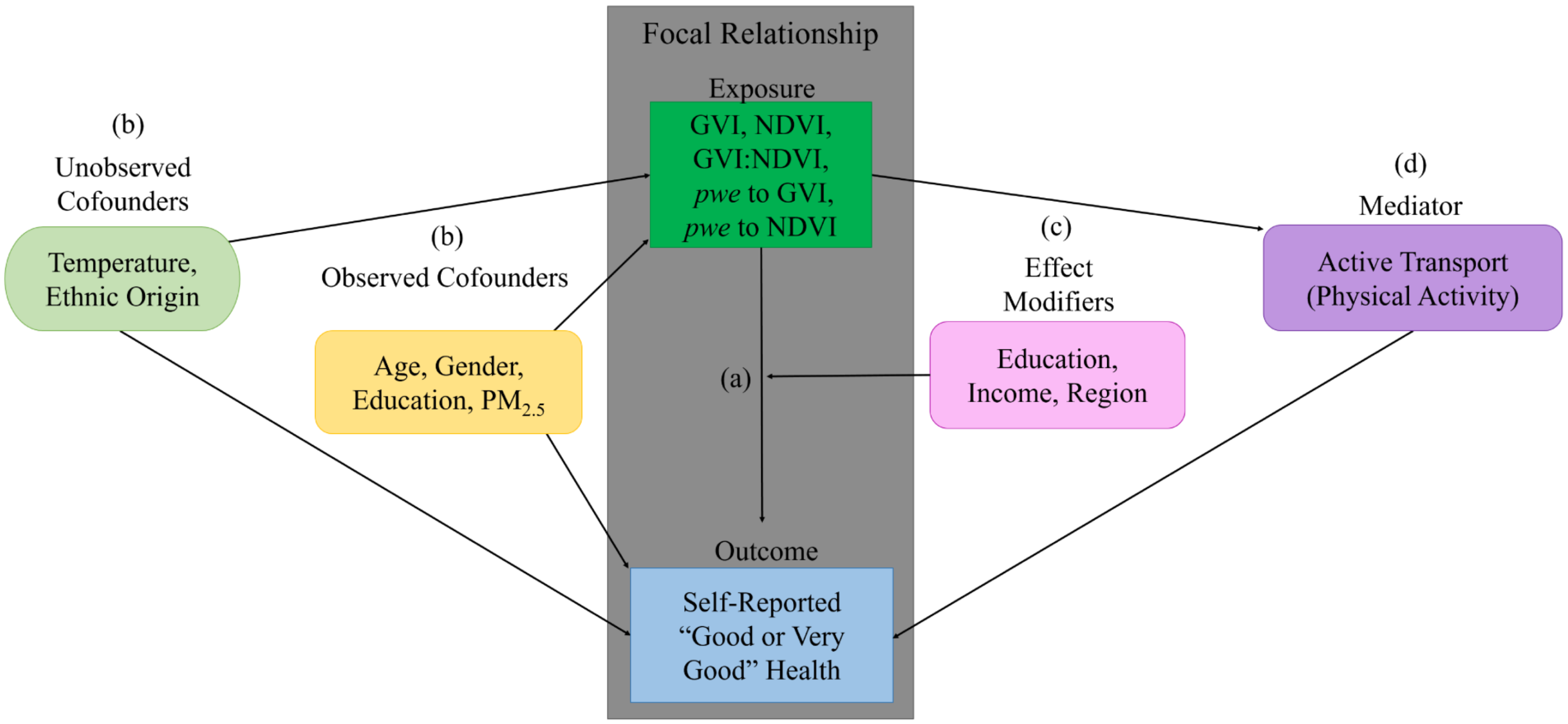

Figure S7. Directed acyclic graph (DAG) demonstrating the relationship between greenspace exposure, self-reported "good or very good" health and their confounders, mediators and effect modifiers. Pathway (a) shows the direct association between greenspace exposure and self-reported "good or very good" health. Pathway (b) shows confounders which are associated with both greenspace exposure and self-reported "good or very good" health. Pathway (c) shows effect modifiers which modify the effect of greenspace exposure on self-reported "good or very good" health. Pathway (d) shows the role of mediators in which greenspace has an indirect effect.
